# ”Being ill was the easy part”: exploring cancer survivors’ reactions to perceived challenges in engaging with primary healthcare

**DOI:** 10.1080/17482631.2024.2361492

**Published:** 2024-06-02

**Authors:** Lars Garpenhag, Anders Halling, Susanna Calling, Linn Rosell, Anna-Maria Larsson

**Affiliations:** a Center for Primary Health Care Research, Department of Clinical Sciences Malmö, Lund University/Region Skåne, Lund, Sweden; b Division of Psychiatry, Department of Clinical Sciences Lund, Lund University, Lund, Sweden; c University Clinic Primary Care Skåne, Region Skåne, Sweden; d Regional Cancer Center South, Lund, Sweden; e Department of Health Sciences, Faculty of Medicine, Lund University, Lund, Sweden; f Division of Oncology, Department of Clinical Sciences Lund, Lund University, Lund, Sweden

**Keywords:** Primary healthcare, cancer survivors, health services accessibility, Sweden, Qualitative research

## Abstract

**Purpose:**

Cancer survivors experience barriers to primary healthcare (PHC) services. The aim was to explore reactions to and opinions about perceived challenges associated with PHC access and quality among cancer survivors in Sweden, including how they have acted to adapt to challenges.

**Methods:**

Five semi-structured focus group interviews were conducted with cancer survivors (*n* = 20) from Skåne, Sweden, diagnosed with breast, prostate, lung, or colorectal cancer or malignant melanoma. Focus groups were mixed in regard to diagnosis. Data were analysed using a descriptive template analysis approach.

**Results:**

In light of perceived challenges associated with access to adequate PHC, participants experienced that they had been forced to work hard to achieve functioning PHC contacts. The demands for self-sufficiency were associated with negative feelings such as loneliness and worry. Participants believed that cancer survivors who lack the ability to express themselves, or sufficient drive, risk missing out on necessary care due to the necessity of being an active patient.

**Conclusions:**

The findings highlight negative patient experiences. They have implications for the organization of care for cancer survivors as they indicate a need for more efficient post-treatment coordination between cancer specialist care and PHC providers, as well as increased support for patients leaving primary cancer treatment.

## Introduction

Cancer survivors—individuals living with or having a history of cancer (National Cancer Institute, n.d.)—experience increased health needs across a spectrum of domains, including physical rehabilitation needs as well as psychological (e.g., fear of recurrence), social (e.g., employment related) and existential (e.g., loss of self) challenges (Henoch & Danielson, 2009; Richardson et al., 2011; Stein et al., 2008). Needs can arise because of cancer and its treatment, at all stages of the clinical trajectory and even long after treatment completion (Wu & Harden, 2015). Together with non-cancer related comorbidities (Earle & Neville, 2004) this results in a potentially complex set of needs (Hoekstra et al., 2014).

The management of cancer survivors’ needs is complicated by increases in cancer incidence and survival, resulting in a growing number of patients requiring the attention of the healthcare system. Globally the number of people diagnosed with cancer is estimated to increase with 47% over the next 20 years (Sung et al., 2021) and in Sweden 100 000 people are expected to be diagnosed with cancer in 2040 (Cancerfonden, 2016). Despite a substantial increase in cancer incidence, mortality is decreasing, rendering an estimated 10-year prevalence of 640 000 by the year 2040 (Cancerfonden, 2016). Accordingly, it has been acknowledged that it is beneficial both to individual and society if primary healthcare (PHC) services are involved throughout the whole cancer care continuum. PHC can play a crucial role not only in the management of non-cancer related comorbidities and health needs, but also in performing basic rehabilitation and treatment follow-up (Blane & Lewandowska, 2019; Earle, 2007). The involvement of PHC in cancer survivors’ care, however, is often challenging, not least due to difficulties surrounding the coordination of cancer specialist care and PHC (Dossett et al., 2017; Handberg et al., 2018).

In Sweden, PHC is allotted a relatively small share of total healthcare resources (Anell, 2015), and, concurrent with international studies on time constraints in the delivery of PHC (Porter et al., 2023), shortage of time in the patient encounter is a common experience among PHC physicians (Socialstyrelsen, 2023). Although national clinical guidelines call for PHC services to provide persons with cancer with preventive and basic forms of care as well as rehabilitation (Regionala cancercentrum i samverkan, 2021), little is known about to what extent this is implemented.

While there are many studies on how people with different cancer forms cope with the impact that the disease and its treatment have on their lives (Lashbrook et al., 2018), their reactions to poor healthcare access or performance have not enticed the same interest. It is known that unsatisfactory relations with healthcare providers evoke negative feelings in people with cancer (Aase et al., 2022; Glasdam et al., 2020; Jefford et al., 2008; Rutherford et al., 2023), and that they employ strategies to manage such relations (Tan et al., 2018). However, there is still a need for exploration of how adverse experiences with health services may affect cancer survivors in the context of different national healthcare systems and branches of healthcare. A recent qualitative study showed that cancer survivors in southern Sweden experience a number of barriers that prevent access to satisfactory PHC services, including deficient communication between cancer specialist care and PHC, and problems tied to lacking availability, continuity, and cancer related competence and engagement among PHC providers (Garpenhag et al., 2023). In the current paper we approach the same data set as in the previous study from a different angle, with the aim to explore reactions to and opinions about challenges associated with PHC access and quality among cancer survivors in Sweden, including how they have acted to adapt to the perceived challenges.

## Material and methods

The study followed a qualitative descriptive design and forms a part of a greater mixed-methods project studying cancer survivors’ experiences with PHC. The study was approved by the Swedish Ethical Review Authority (file no. 2021–03534).

### Setting

The study was conducted in Region Skåne, a county region in southern Sweden. Swedish healthcare is predominantly tax-financed and universal, offering comprehensive services to residents for subsidized fees. Healthcare is decentralized, with each county council being responsible for services in their region (Janlöv et al., 2023). Cancer care is organized according to a national strategy launched by the government in 2009 with the goal to assure equal and high-quality cancer care to all residents (Socialdepartementet, 2009). Highly specialized treatment is provided by the university hospitals, while county hospitals provide more basic treatment.

The PHC sector—based mainly around the primary healthcare centres (PHCCs)—provide a broad range of outpatient care and is supposed to form a pathway to secondary healthcare (non-primary speciality healthcare, e.g., surgery or oncology services) (Blomqvist & Winblad, 2001). The healthcare system is currently undergoing a transition towards a greater focus on primary healthcare, to meet the challenge of serving an ageing population (Samordnad utveckling för god och nära vård, 2018).

### Study participants and data collection

Data were collected through digital focus group interviews, conducted in late 2021 and early 2022. For study inclusion and exclusion criteria, see Table 1. Recruitment took place through advertisements spread digitally by six patient advocacy groups (PAGs) representing people with the relevant diagnoses. The PAGs were approached by the project group with information about the study and a request for help with the advertisement. Key persons from the PAGs were offered follow-up contacts to ensure that they had the information they needed. Depending on the routines and communication infrastructure of each PAG, the advert was spread through mailing lists, posts in social media and through the organizations’ web pages. The interviews, conducted in Swedish using Zoom Meetings (Zoom Video Communications, Inc.), were moderated by the first author. The second, third and last authors rotated in the role as assistant interviewer.

**Table 1.** Inclusion and exclusion criteria.

Inclusion	Exclusion
* Age 18 +	* Terminal phase cancer
* Self-reported initiated treatment for breast cancer, prostate cancer, lung cancer, colorectal cancer or malignant melanoma	* Non-Swedish speaker

Eleven men and 9 women were interviewed (see Table 2 for participant characteristics), in 5 groups each consisting of 3–5 persons. As expected, since we employ an inclusive definition of who is to be considered a cancer survivor, including people with cancer “from the time of diagnosis until the end of life” (National Cancer Institute, n.d.), the sample was heterogeneous regarding time since diagnosis (<1–10 years), treatment received, and positions in the cancer care trajectory. The interviews were semi-structured and based on questions focused primarily on participants’ health needs and experiences of PHC services. Group composition was diverse in respect to gender and diagnosis. Interview audio was recorded and transcribed in its entirety. All participants received information about the study and gave their written informed consent to participate via mail before being interviewed. For more detailed information about the recruitment and data collection procedure, including the interview questioning route, see Garpenhag et al (Garpenhag et al., 2023).

**Table 2.** Participant characteristics.

Characteristics	n	Years
*Gender*		
Male	11	
Female	9	
*Age*		
Min		48
Max		78
Mean		62.5
*Diagnoses*		
Colorectal cancer	8	
Prostate cancer	5	
Breast cancer	3	
Malignant melanoma	3	
Lung cancer	1	

### Analysis

The data were analysed thematically to create a qualitative description of participants’ experiences and views (Sandelowski, 2000), employing template analysis (King, 2004) to provide a framework for the coding and construction of themes. A flexible approach to qualitative data analysis, template analysis revolves around reiterative hierarchical coding of data, and the organization of codes in a template (King, 2004). The first and third authors independently carried out initial coding of a data subset, guided by the study aim but otherwise approaching data inductively. At a subsequent meeting, they compared their coding and, based on consensus, drafted a merged preliminary template organizing codes and themes from the analysed interviews. The preliminary template was then reiteratively refined by the first author in the continued analysis, resulting in the addition of new codes and minor changes in structure to reflect the complete data set. Finally, it was modified based on input from the co-authors before the conclusion of the analytic process.

Quotes in the results section were translated and edited for clarity by the first author. All respondent names are fictitious.

## Results

The findings are organized into three themes, each containing several sub-themes (for an overview, see supplemental material). The first theme, *to be a patient with cancer is hard work*, centres on participants’ accounts of how they have acted to overcome challenges and enhance their contacts with PHC. The second theme concerns *reactions to challenges in accessing adequate PHC*, i.e., the thoughts and feelings that perceived difficulties in accessing PHC had evoked in participants. The final theme, *prerequisites for the successful patient*, concerns the notion that cancer survivors’ chances to secure needed PHC resources are shaped by certain individual characteristics and abilities.

### To be a patient with cancer is hard work

Considering contacts with PHC—and in some cases other healthcare services as well—participants expressed that it takes self-reliance and activity to make them work. What this meant, however, differed between participants, spanning from the meeting of relatively moderate expectations on patient action to the shouldering of great responsibility for the healthcare encounter.

#### Even moderate expectations can appear burdensome

A few participants described even basic activities, which could be considered generic core parts of the patient role, as burdensome work. This included having to independently reach out to PHC services when in need, or to initiate discussions with practitioners about changes in treatment. The notion was raised that if you are ill or exhausted already from the start, this negatively affects the ability to act independently.

It is very much a matter of […] being able to seek care yourself, you know, ask for help. […] Both when it comes to, you know, psychological support and assistance with aids and other stuff, you don’t know … not everyone knows where to call, and it’s not always easy to get hold of that information and often you sit there paralyzed and don’t know how to [do it]. Berit (lung cancer)

#### Struggling for one’s cause

Others described their attempts to acquire adequate help from PHC services as a struggle that demanded great self-reliance. Several participants had felt forced to be stubborn and persuasive to make themselves heard. Others had felt compelled to take a direct and very active role in the management of their health, e.g., thoroughly researching their condition or organizing their own health promotional activities, to make up for perceived non-action on the part of their PHC providers.

I was on my own and had believed that I would get some professional help to investigate [my condition] […] but I had to do it on my own from the beginning and read up on it. [—] I studied and found a drug that should be beneficial for me in this situation, and then I brought in a note to the primary healthcare center, with copies of studies […], and asked to get a drug that they had prescribed there. Robert (colorectal cancer)

#### Acting as a liaison

One particular role that participants felt they had been forced to shoulder was as healthcare liaisons. Due to deficient communication between hospital and PHC professionals, exacerbated by the use of separate electronic record systems, it had been up to the patients to manage and transfer information. To serve this function was experienced as tedious and required the patient to take measures to memorize and keep the facts straight.

You end up as some kind of traffic controller between the different system functionalities, which you’re not supposed to be, and this happens when you are hospitalized as well. It is not as if the hospital department gets in contact with the primary healthcare center directly. John (colorectal cancer)

#### Taking one’s business elsewhere

To some participants, faced with what they believed to be unacceptably low levels of availability or commitment to their care, activity meant voting with their feet by relisting with another PHC provider.

I will change my primary healthcare center, I will, because I can’t take this anymore. I need to move forward and I’ve tried to change [provider], but you weren’t allowed to do that during [treatment]. [—] So that’s what I’ll do now. I hope I’ll end up in a better place. Mona (breast cancer)

Some circumvented the PHCC altogether, substituting it with other services. One participant had stopped going to the PHCC for blood work, and instead visited a hospital test unit which he found to be more available. In addition, several participants had made use of resources outside of formal healthcare. Social media peer groups as well as friends and family in the healthcare professions were mentioned as go-to places for advice.

Then there was the primary healthcare center and … yes, perhaps it was a bit like I didn’t have that connection. Because if I’d been worried or wondered about anything, I feel that [the PHCC] would have been the place to turn to. But I don’t think I really did that all the time. I have many friends who are working in healthcare who … Well, I could take things with them as well. Jonas (colorectal cancer)

#### Reactions to challenges in accessing adequate PHC

While participants did not reject the importance of self-reliance or activity per se, the poor experiences they recounted, and the resulting belief that it was up to patients themselves to make their PHC contacts work, evoked a host of negative thoughts and feelings.

##### Abandonment and worry

A recurring topic was feelings of abandonment. Several participants expressed that they had felt lonely or forgotten about since discharge from cancer specialist care. Feeling that they had nowhere to turn to, some participants were disappointed that PHC services hadn’t been there for them when they needed them.

I’ve felt a bit excluded or forgotten […]. I experienced some mental issues afterwards, and then you are supposed to seek care entirely on your own, and if you do not succeed, there is no one checking on you to see why you have failed. And there I feel that I haven’t received the support that I might have wanted. Anna (colorectal cancer)

Others were worried about how they would be able to manage their healthcare needs, e.g., in case of cancer recurrence. The sources of concern included a lack of efficient contact pathways to PHC that made participants uncertain that they would be able to get help when they needed it, but also a distrust in PHC professionals’ competence in cancer-related matters. One manifestation of the latter was a felt need to be constantly vigilant to ensure that professionals had not overlooked crucial information.

There is this fear that it will take half a year to get to the dermatologist clinic [after initiating contact with primary healthcare]. […] Now when I no longer have the contact nurse to speak to—whom I thought was wonderful—yes, well, who do I speak with then, the mirror perhaps? Ruth (melanoma)

##### Opinions about unfairness

Participants raised matters of principle as well, highlighting the fact that clinical guidelines had not been followed, or reflecting that the amount of responsibility put on them as patients was disproportionate or unfair. Given their oftentimes fragile health, one of them pointed out, it seemed even less fair that they would have to make up for problems that weren’t theirs to solve.

This is how it is, [moderator]. If you want to survive the cancer you don’t have many options but to fix and mend things yourself. […] It shouldn’t be us patients acting as the glue between different healthcare functions, and we aren’t in a particularly good shape when we’re forced to do it either. [But] it is our lives that are at stake. John (colorectal cancer)

##### Contrasts to cancer care

Even though the interviews focused on experiences related to PHC, occasionally participants expressed negative feelings due to problems in other parts of the healthcare system as well. However, for the most part, they described their contacts with cancer specialist care, especially during periods of active treatment, as well-functioning. As cancer patients they had been embedded in a system where they knew whom to turn to and could count on reaching them. Satisfaction with these relationships clearly shaped how participants perceived other healthcare contacts, including those with PHC. In reference to the process of leaving cancer treatment, several participants voiced different variations of the notion that it had been relatively easy to be ill, but all the harder to recover. Practical aspects of the transition (e.g., lost healthcare contact) appeared as part of a wider spectrum of problems associated with survivorship, intertwined with existential issues actualized by recovery (e.g., lost illness identity).

Being ill was the easy part, it is recovery that has been hard. Just because, then you have nothing, no contacts in the healthcare system, and you don’t have, like, a cancer identity anymore. You’re just a regular person again. Jonas (colorectal cancer)

##### Prerequisites for the successful patient

Some participants expressed that, while personally, they had been able to adapt to challenging PHC contacts, this had only been possible because of privilege in different respects, implying that rather than being equally distributed, cancer patients’ chances to satisfy their healthcare needs are dependent on personal characteristics.

###### Language skills

Having sufficient language skills, and being able to express yourself, was considered essential. In this sense, both people with cognitive impairments and non-Swedish speakers were implied to form disadvantaged groups, at risk of not being able to properly protect their interests.

[The satisfaction of health needs] hinges on the patient’s ability to convey [what she needs], that I have been able to tell them. And I’m so grateful that I had the knowledge and, also, that I was able … was able to speak myself, so to say. It would be so difficult, I believe, if there were a language barrier or something like that. Karin (breast cancer)

###### Stamina and drive

Another factor believed to affect patients’ chances was the possession of a drive—variously referred to as stamina, inner strength or energy—necessary to carry on when overwhelmed by adversity. Again, experiences of severe illness and treatment side-effects were brought up as complicating factors, affecting the ability to muster the strength necessary to struggle with healthcare providers. Some construed this as a matter of personality traits. Having an energetic or enterprising personality was described as helpful in moving the healthcare process forward, as was traits such as persistence and the habit to speak up for oneself.

There is no ownership in the background, there is no … it all boils down to you having the energy to make things happen yourself. If you don’t have that, I understand that it goes horribly wrong. And that you’re totally left out. Börje (melanoma)

### Discussion

In this study, we have explored cancer survivors’ reactions to and opinions about challenges in their contacts with PHC services, including how they have acted to adapt to the perceived challenges. The analysis illustrates how patients’ experiences of deficiencies in their relations with healthcare providers risk not only to form a barrier for the utilization of necessary healthcare resources but also to negatively affect patients psychologically, by making them feel abandoned and by casting doubts on their chances to secure necessary help at what already marks a particularly vulnerable time in their lives. The findings are of great relevance to ongoing efforts to reform Swedish healthcare with an emphasis on quality, proximity, and person-centred care (Samordnad utveckling för god och nära vård, 2018).

The finding that cancer survivors may feel obliged to engage in activities they experience as burdensome and unwarranted to manage their PHC contacts and needs corresponds to what has been found in previous studies in different countries (Aase et al., 2022; Handberg & Maribo, 2020; Lawn et al., 2017; Rutherford et al., 2023). Active management of one’s health is a known way to cope with having a cancer diagnosis (Beynon et al., 2015). However, for persons with cancer as well as those suffering from chronic conditions in general, the concerted efforts required to successfully manage one’s healthcare contacts, or to adhere to treatment, risk to turn into a serious burden, with its’ own adverse effects on health (Blane & Lewandowska, 2019; Koch et al., 2015). While patient participation in the shaping of healthcare is desirable, for it to form a constructive element in clinical practice it needs to be clearly defined and based on a well-working relationship between patient and provider (Nilsson et al., 2019). Study participants’ willingness to engage with matters related to their health could prove a valuable resource in the co-creation of care, but the unwanted activities they criticized should thus not be confused with fruitful patient participation.

Because of the disruption in the patient—provider relationship at the end of cancer treatment, the period afterwards seemed to have formed a particularly sensitive phase for several participants. It is well known that many cancer survivors experience this period as difficult, and the negative feelings expressed over non-functioning PHC by participants in this study are echoed in a growing international literature on cancer survivors’ post-treatment situation (Aase et al., 2022; Fitch et al., 2020; Glasdam et al., 2020; Jefford et al., 2008; Llewellyn et al., 2019). Far from it being the sole source of negative feelings, it is reasonable to view dysfunctional PHC experiences as a factor that may aggravate difficulties tied to already present post-treatment distress, exhaustion, confusion, and fears of recurring cancer (Fitch et al., 2020; Glasdam et al., 2020; Sun et al., 2014). Perceptions of post-treatment care must also be viewed in relation to what has preceded it. Our analysis suggests that in Sweden, as in many other places (Aase et al., 2022; Fitch et al., 2020; Jefford et al., 2008; Llewellyn et al., 2019; Sun et al., 2014), positive experiences of cancer specialist care set a baseline that colours cancer survivors’ perceptions of what comes next.

Based on the belief that PHC availability and quality hinge on patients’ language skills and stamina, several participants raised the question of what this means for patients who are not proficient or strong enough to succeed in attempts to access the healthcare they need. Similar concerns have been expressed by cancer survivors regarding access to survivorship care in Denmark, another Scandinavian welfare state with universal healthcare (Handberg & Maribo, 2020). This is an issue with implications for both patient safety and healthcare equity.

The demands for self-reliance and active involvement that participants described, as well as the worry caused by unclarities regarding navigation of the healthcare system, imply a need to strengthen the coordination and communication between healthcare providers, both to bridge gaps in care transitions and to enhance information sharing (Adam & Watson, 2018). Internationally, interventions including organizational models for cooperation between cancer specialist care and PHC (Nekhlyudov et al., 2017) and the use of survivorship care plans (Hewitt et al., 2007) have been suggested as means to increase continuity of care for cancer survivors. Inclusion of PHC representatives in the creation of individual care plans at the beginning of cancer treatment could well act to lessen the gap between services in the Swedish setting. In addition, although it is not yet widely implemented, national Swedish guidelines already call for the use of active handovers of patients to PHC at the conclusion of cancer treatment (Regionala cancercentrum i samverkan, 2021), another type of measure that could strengthen provider coordination.

Participants’ accounts also highlight the importance of strong patient self-efficacy in interactions with healthcare services, that is, that patients believe in their ability to achieve a desired outcome of the contacts (Bandura, 1978). While several participants had been able to overcome the challenges that they had encountered to have their PHC needs met, this was not the case for everyone. One strategy that has been suggested to strengthen the self-efficacy in patients with chronic conditions has been the promotion of health literacy (Lee et al., 2016; Osborn et al., 2010). Health literacy “entails people’s knowledge, motivation and competences to access, understand, appraise, and apply health information” (Sørensen et al., 2012). The kind of difficulties brought up by participants in this study tied to linguistic skills and cognitive and physical abilities are known to influence the level of health literacy and in the extension the consumption of healthcare services and health outcomes (Rajah et al., 2018; Sørensen et al., 2012). Research reports low awareness of the concept among health care professionals, suggesting that educational initiatives (Rajah et al., 2018) could be a fruitful avenue to support high-quality communication between healthcare providers and patients (Nutbeam & Lloyd, 2021). In addition, access to sufficient support that increases patients’ ability to process and use the information are measures that could strengthen health literacy (Liu et al., 2020) and thereby also self-efficacy and patient autonomy.

#### Limitations

One limitation in the current study is that participants were recruited through PAGs. People engaged in PAGs can be expected to be more resourceful, healthier and thus more ready to shoulder an active patient role than other cancer patients. That interviews were conducted in Swedish excluded non-Swedish speakers, and may also have made participation less appealing to non-native Swedish speakers. We acknowledge that a broader representation could have added vital perspectives, not least concerning issues tied to equity. Another potential limitation is that interviews were conducted using videoconference technology, instead of at physical meetings. Even though there are differences between digital and physical meetings, however, studies have found similarities in terms both of focus group interaction (Henage et al., 2021) and the data produced (Woodyatt et al., 2016). Not least, the digital format makes participation easier for people in poor health (Rupert et al., 2017), a benefit that was made crucial by the fact that data collection took place during the COVID-19 pandemic. Finally, it is worth noting that the interviews were centred on the person with cancer as an individual. Although the analysis stands as is, if questions would have been routinely asked about social support from family and friends, this could have provided data for a richer analysis of the meaning of informal support in meeting challenges in PHC access.

#### Future research

Research focusing on the role of family and friends in cancer survivors’ contacts with PHC services could yield a more comprehensive understanding of the dynamics of these contacts. In addition, further qualitative research into PHC clinicians’ perceptions and experiences of providing care to cancer survivors, especially during the post-treatment phase of survivorship, would provide an important complement to the analysis in the present paper. While the topic has been studied quite extensively in some other national contexts (Dossett et al., 2017), it remains unknown how Swedish PHC professionals perceive their role in survivorship care.

## Conclusions

Perceived challenges associated with PHC access and quality make cancer survivors feel a requirement to be self-sufficient and work themselves to manage their health and achieve functioning PHC contacts, and this is associated with negative feelings. The perception among cancer survivors that they have to be resourceful to get adequate support highlights potential problems tied to health equity. The findings have important implications for the organization of cancer survivor care in Sweden, especially post-treatment, as they indicate a need for improved coordination between cancer specialist care and PHC, as well as improved support in the transitional phase that follows on primary treatment.

## Supplementary Material

Overview of themes and sub themes.docx

## Data Availability

The data that support the findings of this study are restricted by the Swedish Ethical Review Authority in order to protect participants’ privacy, but may be available from Susanna Calling (susanna.calling@med.lu.se) upon reasonable request.
