# Supplementary material for: ”Being ill was the easy part”: exploring cancer survivors’ reactions to perceived challenges in engaging with primary healthcare
Source: Int J Qual Stud Health Well-being. 2024 Jun 2;19(1):2361492. doi: 10.1080/17482631.2024.2361492 (PMC11146241; doi:10.1080/17482631.2024.2361492)
Supplement: Overview of themes and sub themes.docx [file ZQHW_A_2361492_SM2203.docx]

‘Being ill was the easy part’: overview of themes and sub-themes

To be a patient with cancer is hard work

*Even moderate expectations can appear burdensome*

*Struggling for one’s cause*

*Acting as a liaison*

*Taking one’s business elsewhere*

Reactions to challenges in accessing adequate primary healthcare

*Abandonment and worry*

*Opinions about unfairness*

*Contrasts to cancer care*

Prerequisites for the successful patient

*Language skills*

*Stamina and drive*
